# Enzyme Treatment-Free and Ligation-Independent Cloning Using Caged Primers in Polymerase Chain Reactions

**DOI:** 10.3390/molecules17010328

**Published:** 2011-12-30

**Authors:** Akinori Kuzuya, Keita Tanaka, Hitoshi Katada, Makoto Komiyama

**Affiliations:** 1 Research Center for Advanced Science and Technology, The University of Tokyo, 4-6-1 Komaba, Meguro, Tokyo 153-8904, Japan; Email: keita@mkomi.rcast.u-tokyo.ac.jp (K.T.); katada@mkomi.rcast.u-tokyo.ac.jp (H.K.); 2 Department of Chemistry and Materials Engineering, Kansai University, 3-3-35 Yamate-cho, Suita, Osaka 564-8680, Japan

**Keywords:** DNA, PCR, caged compounds, ligation, cloning

## Abstract

A new simple scheme for constructing recombinant vectors that does not require any restriction enzyme, ligase, or any other special enzyme treatment has been developed. By using caged primers in PCR, unnatural sticky-ends of any sequence, which are sufficiently long for ligation-independent cloning (LIC), are directly prepared on the product after a brief UVA irradiation. Target genes and vectors amplified by this light-assisted cohesive-ending (LACE) PCR join together in the desired arrangement in a simple mixture of them, tightly enough to be repaired and ligated in competent cells.

## 1. Introduction

In current molecular biology and biotechnology, vectors are constructed by digesting plasmid DNA with restriction enzymes, followed by connection of this vector with predetermined gene fragment using ligase. In 1990, Aslanidis and de Jong proposed a system that may dramatically simplify this process [1]. In this ligation-independent cloning (LIC) of PCR products, unnaturally long sticky ends of around 10–12-mer are formed on the ends of both a vector and a target gene fragment. Even if they are not ligated *in vitro*, simple mixing of these fragments gives a quite stable vector-insert complex that can come into competent cells, and let the nicks be repaired by cells’ inherent repair pathways. The most important advantage of LIC is that it can eliminate any need to use restriction enzymes, which basically allows any gene to be cloned regardless of its sequence. To date, several methods to provide such unnaturally long sticky-ends to PCR products have been reported [1,2,3,4,5,6]. However, most of these techniques still require special design of sticky ends, additional time and budget-consuming enzymatic processing of PCR products, which often limit the scope of applications of LIC.

**Scheme 1:**
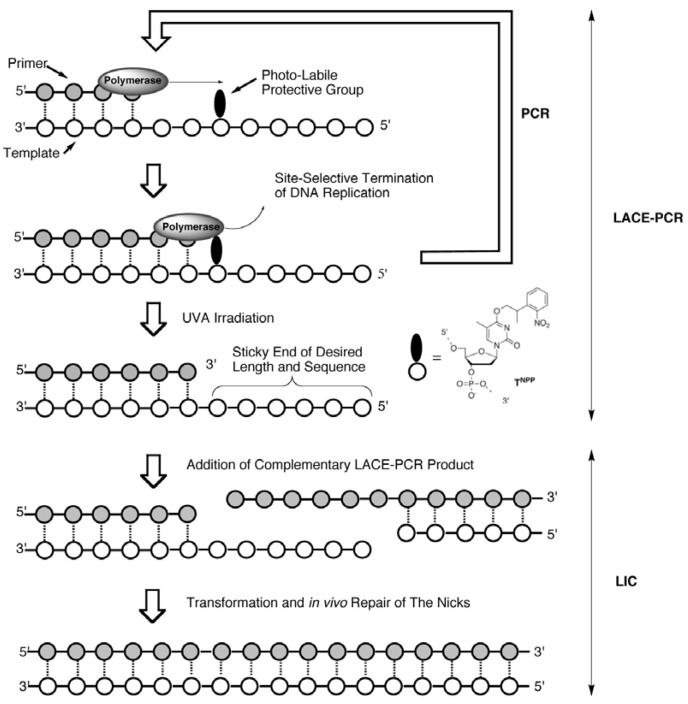
A schematic illustration of LACE-PCR and LIC.

Recently, we have developed a quite simple and effective new chemistry-based PCR system, Light-Assisted Cohesive-Ending (LACE) PCR, that directly gives product with any desired sticky ends on both its ends (Scheme 1) [7,8,9]. In order to terminate a polymerase reaction at a desired position, a protected nucleotide with a photolabile protective group (a caged nucleotide) is incorporated into PCR primers [10]. Within PCR cycles, elongation of the nascent strand (5′→3′ direction) is site-selectively terminated at the 3′-side of the caged nucleotide. Accordingly, the 5′-portion in the primer from the caged nucleotide remains single-stranded throughout the cycles, and predetermined sticky ends are obtained after the removal of the protecting group by brief UVA irradiation. No enzyme treatment on PCR product is necessary for the preparation of sticky-ends in this system.

The usefulness of LACE-PCR has been shown first in a conventional cloning using ligase with a sticky-ended plasmid prepared with restriction enzymes and an insert prepared with LACE-PCR [8,9], and recently in introducing point mutation in a plasmid as a variation of QuickChange^TM^ technique [11], which involves simple self-circularization of sticky-ended products [12]. Potential difficulties have been expected, on the other hand, in the application of LACE-PCR to LIC. For example, incomplete photo-removal of the caging group inhibits nick repair in cells exponentially with the number of the fragments to be joined. Possibility of DNA damages induced by UVA also increases as longer LACE-PCR products are required for a vector fragment [13,14,15,16,17,18].

In this study, we have examined various types of recombination including a construction of a gene encoding green fluorescent protein (GFP)-blue fluorescent protein (BFP) fusion protein, and confirmed that LIC is successfully achievable with LACE-PCR products, despite the above potential difficulties. 

## 2. Results and Discussion

**Scheme 2:**
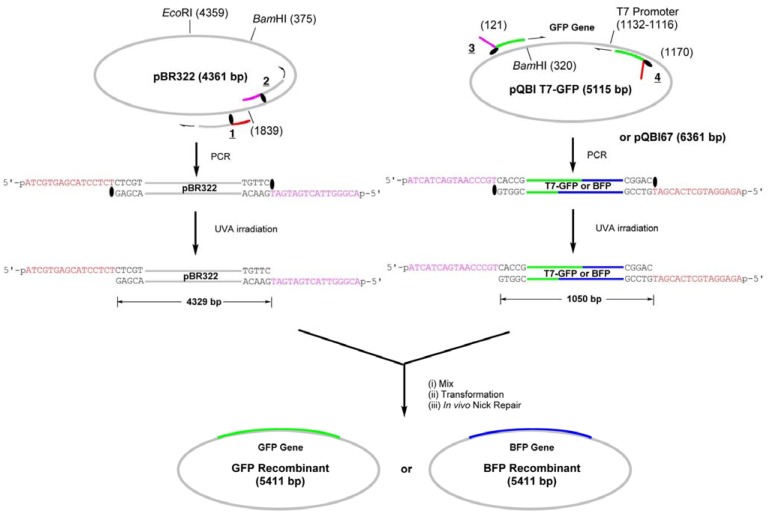
Construction of GFP- or BFP-recombinant by LIC of LACE-PCR products.

We have first constructed simple GFP-recombinant vector by amplifying the whole strand of pBR322 plasmid by LACE-PCR and inserting GFP gene, which is amplified from pQBI-T7GFP vector, into it (Scheme 2).

**Figure 1:**
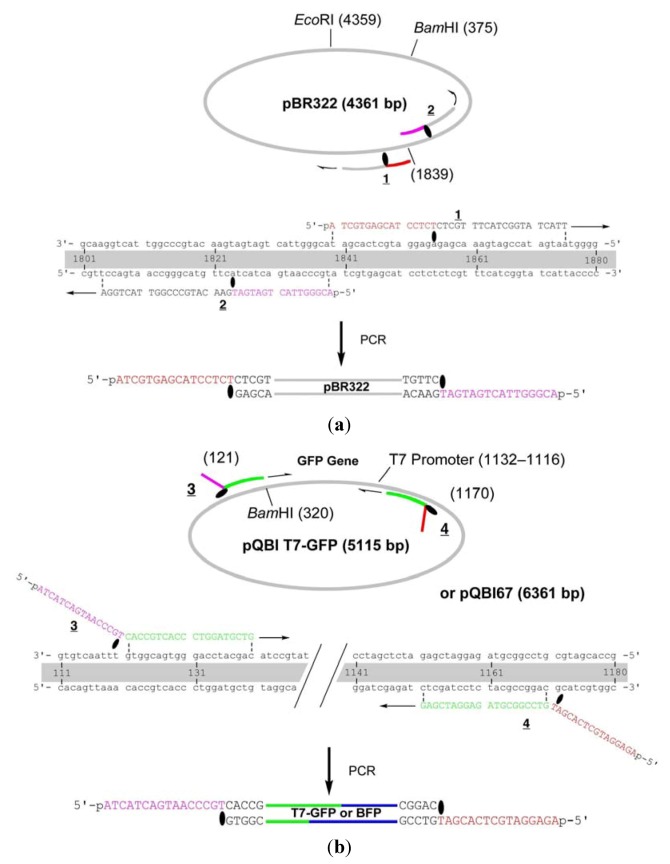
Detailed structure of the primers for the construction of GFP- and BFP-recombinants. (**a**) Primers for the vector fragment;(**b**) Primers for the insert.

The primers **1** and **2** are complementary to pBR322 and used to amplify the whole strand of the vector (Figure 1). These primers were chemically 5′-phosphorylated for the purpose of aiding repair in the cells, although it turned out in the construction of the fusion recombinant that the 5′-phosphates are not essential in the process (see below). The primer **1** hybridizes to 1840–1875-bp region and a caged thymidine, 4-*O*-(2-(2-nitrophenyl)propyl)thymidine developed by Heckel *et al*. (T^NPP^ in Scheme 2) [19] is incorporated to the position of T1855. This primer is completely complementary (except for the NPP introduction) to the plasmid, but the polymerase reaction in PCR is site-selectively blocked by the T^NPP^ [7].

Thus, the complementary part of the 15 nt in the 5′ side of T^NPP^ is not synthesized, and this portion serves as 16-nt sticky end after LACE-PCR. The primer **2**, which has T^NPP^ as the 16th nucleotide, binds to 1804–1839-bp region and similarly produces 16-nt sticky end with the sequence of 1824–1839-bp region. GFP gene was amplified by using **3** and **4**, which are designed to hybridize to 121–140-bp and 1151–1170-bp regions in pQBI T7-GFP. The product obtained with these primers contains T7 promoter, open reading frame (ORF), and T7 terminator. To the 5′ end of **3**, 16-nt stretch containing T^NPP^ and complementary to the sticky end in **2** is attached. Similarly, 16 nt complementary to the sticky end in **1** is attached to the 5′ end of **4**.

**Figure 2:**
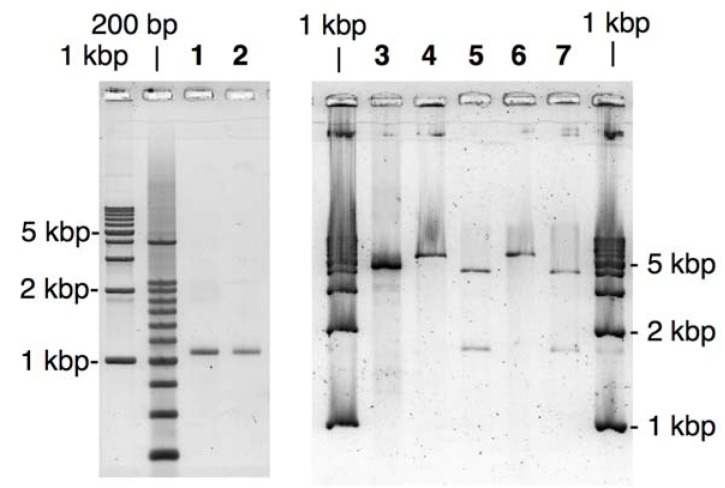
Agarose-gel analysis of LACE-PCR products and the extracted recombinant plasmids for GFP- and BFP-recombinants. Lane 1, GFP fragment; Lane 2, BFP fragment; Lane 3, the vector fragment; Lane 4, GFP recombinant linearized with *EcoRI*; Lane 5, GFP recombinant treated with *BamHI*; Lane 6, BFP recombinant linearized with *EcoRI*; Lane 7, BFP recombinant treated with *BamHI*.

PCR amplification of the fragments was performed by using *Pfu* polymerase, one of the most popular high-fidelity polymerases. The product was purified with a commercially available kit as Tris-EDTA (TE) buffered solution (pH 8.5), and then irradiated with UVA (300 < λ < 400 nm) for 15 min to remove NPP. As shown in Figure 2, fairly pure products of expected length were obtained with the above procedure for both of GFP- and the vector fragments.

Transformation of *E. coli* using these LACE-PCR products was performed without any ligation process as follows: the GFP fragment (2 molar eq. ratio) was simply added to the solution of the vector fragment and incubated for 1 h at 37 °C. This mixture was then used for standard transformation of JM109 competent cells. Note that no enzymatic or chemical treatment was performed here. After overnight incubation at 37 °C, more than 100 colonies were obtained on an agar plate containing carbenicillin. Randomly selected 16 colonies were subjected to colony direct PCR using primers complementary to pBR322, 200 bp away from each of the joints, and eight of them gave the expected 1.4-kbp product from the desired GFP recombinant.

Construction of the recombinant coding BFP gene was performed in a same manner using the LACE-product from pQBI67. Within 16 colonies tested by direct PCR, 5 colonies were positive. Lanes 4–7 in Figure 2 show an analysis of the extracted plasmid from the positive colonies. The vector fragment amplified by LACE-PCR contains one *Eco*RI and one *Bam*HI cleavage site, and both of the GFP and BFP fragments contain one *Bam*HI site. If the complex of the vector and the insert fragment is successfully repaired in *E. coli* and the desired recombinant vector is formed there, the extracted plasmid should give one linearized band of 5.4 kbp after *Eco*RI digestion and two fragmented bands of 1.6 kbp and 3.8 kbp after *Bam*HI digestion. All of the observed bands in Figure 2 are consistent with this argument. Moreover, sequencing of the extracted 10 plasmids has clearly shown that the nick between the two fragments were properly repaired in *E. coli* and no mutation or deletion was induced around the joints and in ORF (see Supporting Information).

**Scheme 3:**
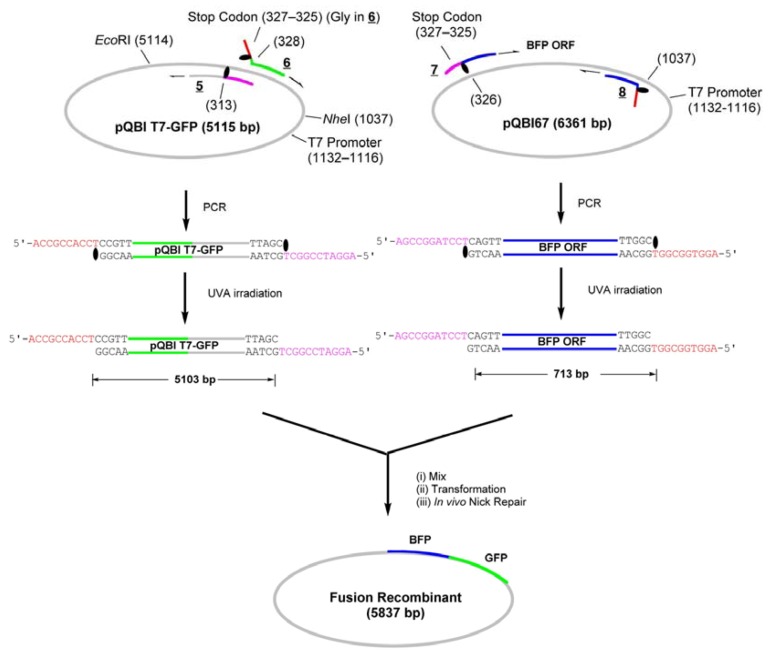
Construction of the fusion-recombinant encoding GFP-BFP fusion protein by LIC of LACE-PCR products.

In addition to the above simple insertion of a gene to a vector, we have examined a possibility of making a fusion protein by using LACE-PCR without ligation. The homology between GFP and BFP is nearly 98%. They may thus be inappropriate substrates for the systems utilizing homologous recombination mechanisms such as In-Fusion technology [6]. For this purpose, ORF of BFP was amplified from pQBI67 and inserted to the end of GFP gene in pQBI T7-GFP (Scheme 3).

**Figure 3:**
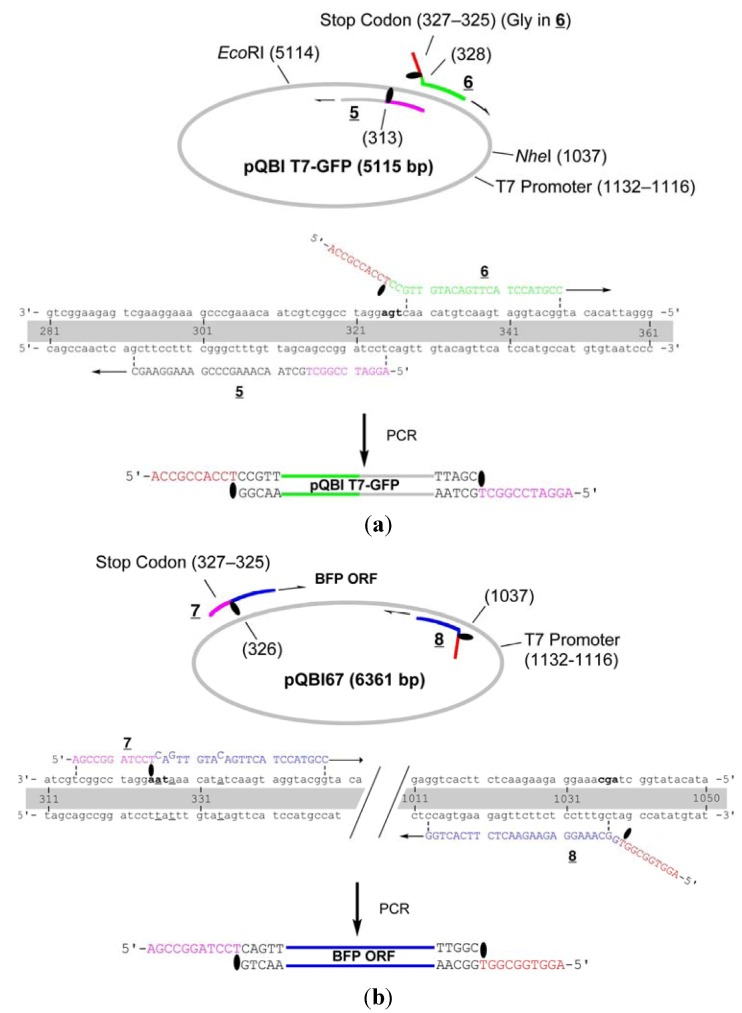
Detailed structure of the primers for the construction of the fusion recombinant. (**a**) Primers for the vector fragment; (**b**) Primers for the insert. The opal stop codon in pQBI T7-GFP, ochre in pQBI67, and the codon for the third amino residue in ORF of GFP (Ser) are shown in boldface. The alteration in the sequence of pQBI67 from that of pQBI T7-GFP (T326. T328, and T334) is underlined.

The whole strand of pQBI T7-GFP except for the 314–327-bp region, which contains an opal stop codon for GFP, were first amplified by LACE-PCR. The caged primers used in this experiment were not phosphorylated. The primers **5** and **6**, which were used to amplify the vector, hybridize adjacent to this omitted region (Figure 3). The primer 6 hybridizes to 291–313-bp region and has T^NPP^ in front of A314. In the 5′ side of T^NPP^, 10-nt stretch with the sequence of 315–325-bp region is added. The primer 5 hybridizes to 328–348-bp region. In this primer, the nucleotide corresponding to A327 in the plasmid is changed to dC in order to mutate the opal stop codon to Gly. In addition, 9-nt stretch that corresponds to Gly ×3 and T^NPP^ is attached to the 5′ end. This portion serves as the sticky end after LACE-PCR, and as a part of Gly ×5 linker between GFP and BFP. Since the only requirement in LACE-PCR is the introduction of single caged nucleotide in a primer, such alteration of amino-acid sequence around the ends of the fragment is quite easy.

**Figure 4:**
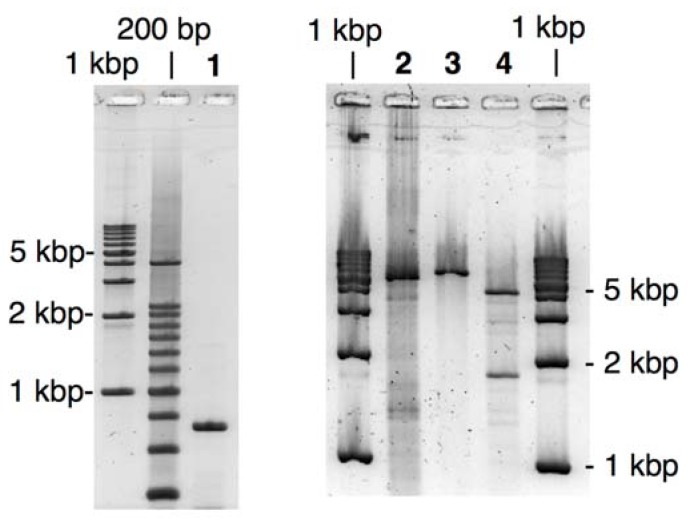
Agarose-gel analysis of LACE-PCR product and the extracted recombinant plasmid for the fusion recombinant. Lane 1, ORF fragment of BFP; Lane 2, the vector fragment; Lane 3, the fusion recombinant linearized with *EcoRI*; Lane 4, the fusion recombinant treated with *EcoRI* and *NheI*.

The primers **7** and **8** are designed to amplify ORF (325–1035-bp region) of BFP coded on pQBI67. The primer **7** hybridizes to 326–348-bp region and has T^NPP^ in front of A325. In the 5′ side of T^NPP^, 10-nt stretch with the sequence of 314–324-bp region is added. Because of the sequence difference between pQBI T7-GFP and pQBI67, T326, T328, and T334 in pQBI67 are mismatched with **7**. With the aid of this 10-nt stretch, however, **7** binds to the template strong enough to give fair amount of the desired product as shown below. The primer **8** hybridizes to 1013–1037-bp region. To mutate the third amino residue in BFP (Ser) to Gly, dG is incorporated in front of T1038 in the plasmid, and 9-nt stretch that is complementary to the Gly ×3 linker attached to **5** is introduced to the 5′ end. The size of the products obtained after LACE-PCR using these primers were all consistent with the design (lanes 1 and 2 in Figure 4).

Transformation of JM109 using the simple mixture of both of these fragments was performed in a similar manner as described above. In direct-PCR test, 80% of the colonies obtained after overnight incubation were positive. This improved yield is probably because of the extended *Dpn*I treatment and the resulting complete elimination of the template plasmid. As shown in Figure 4, the extracted recombinant plasmid linearized with *Eco*RI (lane 3) was significantly longer than the LACE-PCR product of pQBI T7-GFP (lane 2). When the recombinant plasmid was double digested with *Eco*RI and *Nhe*I, two bands with the expected mobility (1.9- and 4.0-kbp fragments) were clearly observed. The sequence around the joints and ORF again completely agreed with the design (see Supporting Information).

**Figure 5:**
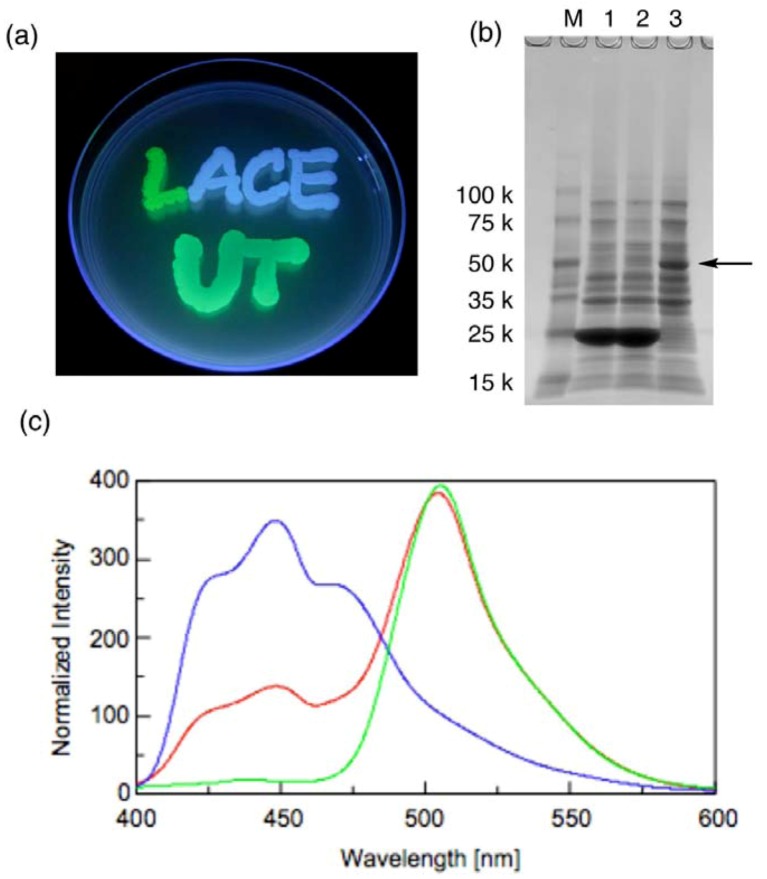
Analyses of expressed fluorescent proteins. (**a**) Fluorescence from the three types of transformed *E. coli*; (**b**) SDS-PAGE of the lysate. Lane 1, Cells transformed with GFP-recombinant; Lane 2, BFP-recombinant; Lane 3, the fusion recombinant; M, size marker; (**c**) Normalized fluorescence emission spectra of the lysate. Green line, GFP-recombinant; blue line, BFP-recombinant; red line, the fusion recombinant. The excitation wavelength is 380 nm.

The extracted plasmids obtained above were further used to transform BL21-Gold (DE3) competent cells, in which T7 polymerase is expressed. Figure 5a shows the emission of green fluorescence from the expressed GFP from the GFP-recombinant (the letter “L”), blue fluorescence from the expressed BFP from the BFP-recombinant (the letters “ACE”) and bluish green fluorescent from the expressed GFP-BFP fusion protein from the fusion recombinant (the letters “UT”) in each of the transformed cells. Successful fluorescence emission from each of the recombinants supports the sequencing analyses. The expressed proteins were further analyzed by preparing lysate of these transformed cells. The size of each protein was confirmed by SDS-PAGE of such lysate (Figure 5b). 

Very dark bands with mobility close to the 25-kDa marker, which is consistent with the expected molecular weight of native GFP and BFP (28 kDa), were observed for the lysate of GFP and BFP recombinants. For the lysate of the fusion recombinant, on the other hand, this band completely disappeared and a new significant band was observed near the 50-kDa marker. Fluorescence emission spectra of the lysate of the fusion recombinant showed two emission maxima at 449 nm and 504 nm, which correspond to the emission from BFP and GFP, respectively (Figure 5c). The emission at 504 nm was significantly stronger than that at 449 nm. Considering that the excitation maximum wavelength of GFP is 473 nm, the large difference of the emission intensity may be attributable to fluorescence resonance energy transfer (FRET) between the two fluorophores, which is another evidence of successful construction of the fusion protein. All of these facts indicate that no critical mutation was induced in the regulatory elements such as promoter, ribosome-binding sites, *etc*. in relatively longer vector fragments as well as ORF in the inserts.

## 3. Experimental

### 3.1. Materials

pBR322 plasmid was purchased from Takara Bio (Japan) and pQBI T7-GFP and pQBI 67 plasmids were purchased from Wako Pure Chemicals (Japan). *Pfu* DNA polymerases were purchased from Promega (USA).

### 3.2. Preparation of Caged Primers

The caged primers were prepared on an automated DNA synthesizer using a set of ultramild cyanoethyl phosphoramidites (phenoxyacetyl protected dA and 4-isopropylphenoxyacetyl protected dG monomers from Glen Research), and purified as described in our previous paper [8]. The phosphoramidite monomer of T^NPP^ was synthesized according to the literature [19].

### 3.3. Light-Assisted Cohesive-Ending PCR (LACE-PCR)

PCR was performed under the following conditions: [template] = 1 ng/µL, [each primer] = 300 nM, [dNTPs] = 200 µM, and [*Pfu* Ultra polymerase] (for the vectors) or [*Pfu* Turbo polymerase] (for the inserts) = 0.05 U/µL, total volume = 50 µL. Thermal cycles: denaturation, 95 °C (30 s); annealing, 55 °C (30 s); elongation, 72 °C (60 s for the inserts and 300 s for the vector fragments). After 30 rounds of thermal cycles, the reaction mixture was treated with *Dpn*I at 37 °C for 1 h (overnight for the fragments for fusion protein) to degrade and prevent any contamination of the template plasmid afterwards. The product was purified with QIAquick PCR purification Kit (Qiagen) as a TE buffered solution (pH 8.5). UVA irradiation on the product was performed for 15 min by using a UV Spot Light Source (Hamamatsu Photonics; 200 W) through a UTVAF-50S-36U filter (Sigma-Koki) at 2.5 mW/cm^2^.

### 3.4. Construction of the GFP-Recombinant

The solutions containing vector fragment (6 ng) and GFP fragment (4 ng) were mixed (final volume, 4 µL) and incubated at 37 °C for 1 h. This mixture was added to the solution of JM109 competent cells (Toyobo, 50 µL), allowed to stay on ice for 30 min, at 42 °C for 40 s, and on ice again for 2 min. To this mixture was added SOC media (500 µL), gently mixed, and shook for 50 min at 37 °C. The mixture was centrifuged at 2000 G for 2 min and the supernatant was discarded. The cells were suspended in the media by gentle pipetting, and spread on LB-agar plate containing carbenicillin (50 µg/mL). After overnight incubation at 37 °C, more than 100 colonies were obtained. Randomly picked 16 colonies were subjected to colony direct PCR using 5′-TGCACCATTATGTTCCGGATCTG-3′ (1651–1673-bp region of pBR322) and 5′-AAGCTCATCAGCGTGGTCGTG-3′ (2058–2038-bp region of pBR322) as the primers, and 4 of them contained the desired recombinant plasmid. The positive colonies were further cultured in LB media. The recombinant plasmid was purified with a QIAprep spin Miniprep Kit (Qiagen), and its sequence starting 200 bp away from each joint was determined on a 3130× Genetic Analyzer (Applied Biosystems) using the same primers for the colony direct PCR (Supporting Figure 1a). 

### 3.5. Construction of the BFP-Recombinant

The solutions containing vector fragment (10 ng) and BFP fragment (8 ng) were mixed (final volume, 5 µL) and incubated at 37 °C for 1 h. This mixture was added to the solution of JM109 competent cells (50 µL), allowed to stay on ice for 30 min, at 42 °C for 40 s, and on ice again for 2 min. To this mixture was added SOC media (500 µL), gently mixed, and shook for 50 min at 37 °C. The mixture was centrifuged at 2000 G for 2 min and the supernatant was discarded. The cells were suspended in the media by gentle pipetting, and spread on LB-agar plate containing carbenicillin (50 µg/mL). After overnight incubation at 37 °C, 18 colonies were obtained. Randomly picked 16 colonies were subjected to colony direct PCR using the same primers for GFP recombinant, and 5 of them contained the desired recombinant plasmid. The positive colonies were further cultured in LB media. The recombinant plasmid was purified and sequenced as the GFP recombinant (Supporting Figure 1b).

### 3.6. Construction of the Fusion Recombinant

The solutions containing vector fragment (50 ng) and BFP fragment (24 ng) were mixed (final volume, 2 µL) and incubated at 37 °C for 1 h. This mixture was added to the solution of JM109 competent cells (50 µL), allowed to stay on ice for 30 min, at 42 °C for 40 s, and on ice again for 2 min. To this mixture was added SOC media (500 µL), gently mixed, and shook for 50 min at 37 °C. The mixture was centrifuged at 2,000 G for 2 min and the supernatant was discarded. The cells were suspended in the media by gentle pipetting, and spread on LB-agar plate containing carbenicillin (50 µg/mL). After overnight incubation at 37 °C, 6 colonies were obtained, and 5 of them contained the desired recombinant plasmid. For the colony direct PCR, 5′-TGCGGGATATCCGGATATAG-3′ (178–199-bp region of pQBI T7-GFP) and 5′-GCGTCCGGCGTAGAGGATCG-3′ (1172–1153-bp region of pQBI T7-GFP) were used as the primers. Because of the high homology between GFP and BFP, one of the primer for the fusion recombinant, is designed to hybridize 800 bp away the GFP-BFP conjunction. The positive colonies were further cultured in LB media. The recombinant plasmid was purified and sequenced using the above primers (Supporting Figure 1c).

### 3.7. Construction of the GFP-Recombinant

In order to analyze the fluorescent proteins, the purified vector was introduced into BL21-Gold (DE3) (Stratagene). The picture of the culture was taken on a UV transilluminator. The lysate of each transformed cells was performed as follows. The cells cultured overnight at 37 °C in LB media (1.5 mL) was centrifuged at 14,000 G for 10 min, and the supernatant was discarded. Then, BugBuster Protein Extraction Reagent (300 µL, Novagen) was added. The mixture was briefly vortexed, rotated for 20 min at room temperature, and centrifuged at 16,000 G for 20 min at 4 °C. The supernatant was collected and used as the lysate without further purification. For SDS-PAGE, the lysate (20 µL) was mixed with loading dye and heated at 90 °C for 5 min. Fluorescence emission spectra of the lysate, which was diluted by one part of Tris buffer (10 mM, pH 8.0), was measured on a JASCO FP-6500 spectrofluorometer with excitation wavelength at 380 nm.

## 4. Conclusions

It is confirmed that chemistry-based new PCR (LACE-PCR) is straightforwardly compatible with LIC. By simply mixing sticky-ended fragments prepared by LACE-PCR, desired recombinant plasmid can be obtained *in situ* in *E. coli*. The connecting sequence between the two fragments can be freely chosen independently from restriction-enzyme sequences or other special treatments, which may allow cloning of any genes into vectors.

## Supplementary Materials

Supplementary materials can be accessed at: http://www.mdpi.com/1420-3049/17/1/328/s1.
