# Supplementary material for: Enzyme Treatment-Free and Ligation-Independent Cloning Using Caged Primers in Polymerase Chain Reactions
Source: Molecules. 2011 Dec 30;17(1):328–40. doi: 10.3390/molecules17010328 (PMC6290560; doi:10.3390/molecules17010328)
Supplement: Supplementary file 1 [file molecules-17-00328-s001.pdf]

Supporting Information for:

"Restriction-Enzyme-Free and Ligation-Independent Cloning Using Caged Primers in Polymerase Chain Reaction"

Akinori Kuzuya, Keita Tanaka, Hitoshi Katada, and Makoto Komiyama

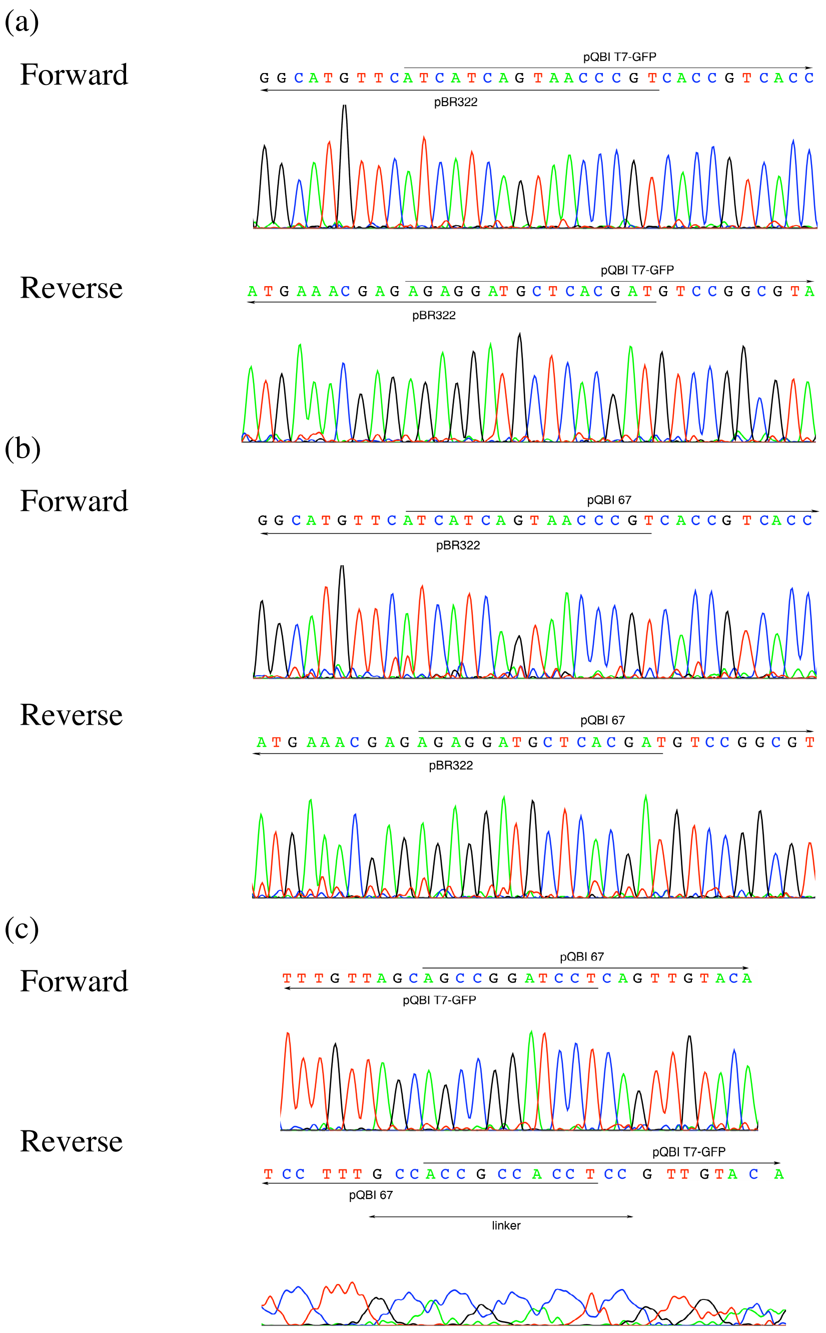

Supporting Figure 1. Sequencing of the recombinant plasmids. (a) A GFP-recombinant. (b) A BFP-recombinant. (c) One of the fusion recombinant.
